# Regulation of psoriasis, colitis, and the intestinal microbiota by clusterin

**DOI:** 10.1038/s41598-023-42019-y

**Published:** 2023-09-16

**Authors:** Yu Kyung Jun, Hee Tae Yoon, So Hyun Kwon, Ui Hyeon Jo, Ji Eun Kim, Yoo Min Han, Min-Seon Kim, Jong Pil Im, Dong Ho Lee, Joo Sung Kim, Seong-Joon Koh, Hyunsun Park

**Affiliations:** 1 Division of Gastroenterology, Department of Internal Medicine, Seoul National University Bundang Hospital, Seongnam, Korea; 2 Present Address: Laboratory of Intestinal Mucosa and Skin Immunology, Liver Research Institute and Seoul National University College of Medicine, Seoul, Korea; 3 Department of Dermatology, SMG-SNU Boramae Medical Center, Seoul, Korea; 4 Department of Pathology, SMG-SNU Boramae Medical Center, Seoul, Korea; 5 Department of Internal Medicine and Healthcare Research Institute, Seoul National University Hospital Healthcare System Gangnam Center, Seoul, Korea; 6 Division of Endocrinology and Metabolism, Department of Internal Medicine, University of Ulsan College of Medicine, Seoul, Korea; 7 Department of Dermatology, Seoul National University College of Medicine, Seoul, Korea

**Keywords:** Skin diseases, Immunology, Gastroenterology

## Abstract

Psoriasis, a chronic and systemic inflammatory disorder characterized by activation of the interleukin (IL)-23/IL-17 axis, may be associated with the intestinal microbiota through the so-called “gut–skin axis.” Clusterin is a glycoprotein ubiquitously distributed in mammalian tissues; however, its role in psoriasis is unclear. Therefore, we evaluated the role of clusterin in psoriatic skin inflammation, systemic inflammation, and colitis using a murine model of IMQ-induced psoriasis. In IMQ-treated clusterin-knockout (*clusterin*^−/−^) mice, the expressions of inflammatory cytokines in clusterin-silenced human keratinocytes and intestinal microbial composition were analyzed. We also examined clusterin expression in the skin tissues of patients with psoriasis. IMQ-induced psoriatic skin inflammation is suppressed in *clusterin*^−/−^ mice. Long-term administration of IMQ induced systemic inflammation and colitis; however, both were alleviated by the genetic deletion of clusterin. Genetic silencing of clusterin in human keratinocytes inhibited the production of inflammatory cytokines involved in the initiation and progression of psoriasis. The composition of the intestinal microbiota in IMQ-treated *clusterin*^−/−^ and wild-type mice was different. Genetic deletion of clusterin suppressed the increase in the Firmicutes/Bacteroidetes (F/B) ratio. Skin tissues of patients with psoriasis showed high clusterin expression. In conclusion, inhibition of clusterin decreased psoriatic skin inflammation, systemic inflammation, colitis, and altered the F/B ratio in an IMQ-induced murine psoriasis model.

## Introduction

Psoriasis is a chronic relapsing inflammatory skin disease caused by the disruption of the innate and acquired immune systems^1,2^. Activation and maturation of dendritic cells (DCs) can trigger skin inflammation. Activated DCs stimulate the proliferation and differentiation of type 1 helper T (Th1) and Th17 cells via tumor necrosis factor (TNF)-α, interleukin (IL)-23, and IL-12. Th17 cells produce IL-17, IL-21, and IL-22 that induce keratinocyte proliferation. IL-17 and IL-23 are key inflammatory cytokines involved in the development of psoriasis. Antagonists of the IL-23/IL-17 axis are used clinically to treat psoriasis^3^.

Imiquimod (IMQ), an agonist of toll-like receptor 7/8, activates the IL-23/IL-17 axis in the epidermis^4^. Topical application of IMQ promotes psoriasis-like skin inflammation. The IMQ-induced murine psoriasis model proficiently reflects human psoriasis, in which the IL-23/IL-17 axis plays a critical role, and is widely used to demonstrate the efficacy of preclinical drugs in treating psoriasis^5^. Moreover, an IMQ-induced psoriasis model using IL-10 deficient (*IL-10*^−*/*−^) mice instead of wild-type (WT) mice, is characterized by the development of severe and persistent psoriatic inflammation^6^.

Multiple environmental factors can induce inflammatory reactions in the skin, leading psoriasis. The gut microbiota, which plays a role in inflammatory skin reactions, is important in food digestion, host immunity, drug/toxin metabolism, and the regulation of gut neuroendocrine function^7^. The gut microbiota composition can change according to the mode of birth, food consumed by infants and adults, lifestyle, and medication. The “gut–skin axis” theory suggests that the gastrointestinal microbial composition has a close relationship with the skin; thus, gut dysbiosis may be linked to dermatologic diseases such as psoriasis, atopic dermatitis, rosacea, and alopecia^8–13^.

Clusterin, also known as an apolipoprotein, is a heterodimeric glycoprotein distributed almost ubiquitously in mammalian tissues. Clusterin is involved in inflammation, autoimmunity, apoptosis, lipid metabolism, neurodegeneration, and tumorigenesis^14,15^. The human clusterin gene is located on chromosome 8p21-p12 and composed of 11 exons. Clusterin transcription produces 3 mRNA isoforms^16^. After translation, the precursor protein of human clusterin folds and forms intramolecular disulfide bonds in the endoplasmic reticulum (ER). After the human clusterin precursor protein is translocated from the ER to the Golgi apparatus, it is glycosylated and cleaved^17^. Mature clusterin is a heterodimeric N-glycosylated protein consisting of an α- and a β-chain linked by 5 disulfide bonds^14^. Clusterin proteins can be either secretory or intracellular form.

The human plasma clusterin level is approximately 20.0–90.0 µg/mL and is positively correlated with systemic inflammation^18^. Clusterin levels in plasma and synovial fluid are associated with the severity of synovial inflammation and cartilage degeneration in patients with osteoarthritis^19^. Clusterin is also related to asthma, a chronic inflammatory airway disorder. Patients with asthma exacerbation display higher clusterin levels in sputum than those with stable asthma^20^. The receptor for clusterin may differ according to the organ, tissue, and cell type. Plenxin A4 is highly expressed in the brain, and acts as a high-affinity clusterin receptor. Decreased levels of plenxin A4 in the brain are related to the aggravation of Alzheimer’s disease^21^. Clusterin can also bind to apolipoprotein E receptor 2 and very-low-density lipoprotein receptors and trigger neuroblast chain formation in the subventricular zone of the brain.

Clusterin expression in keratinocytes is associated with epithelial differentiation and stratification^22,23^. HaCaT cells, immortalized human keratinocytes, also produce clusterin^24^. Serum clusterin levels are higher in patients with psoriasis than in healthy controls^25,26^, and the clusterin mRNA level in skin lesions from patients with psoriasis is different from that in normal controls^27^. However, little is known about how clusterin affects the inflammatory reactions associated with psoriasis. Therefore, we examined the role of clusterin in skin inflammatory reactions in an IMQ-induced murine psoriasis model and in human patients with psoriasis.

## Results

### Genetic deletion of clusterin reduces psoriasis-like inflammatory reactions induced by short-term IMQ administration

**Figure 1:**
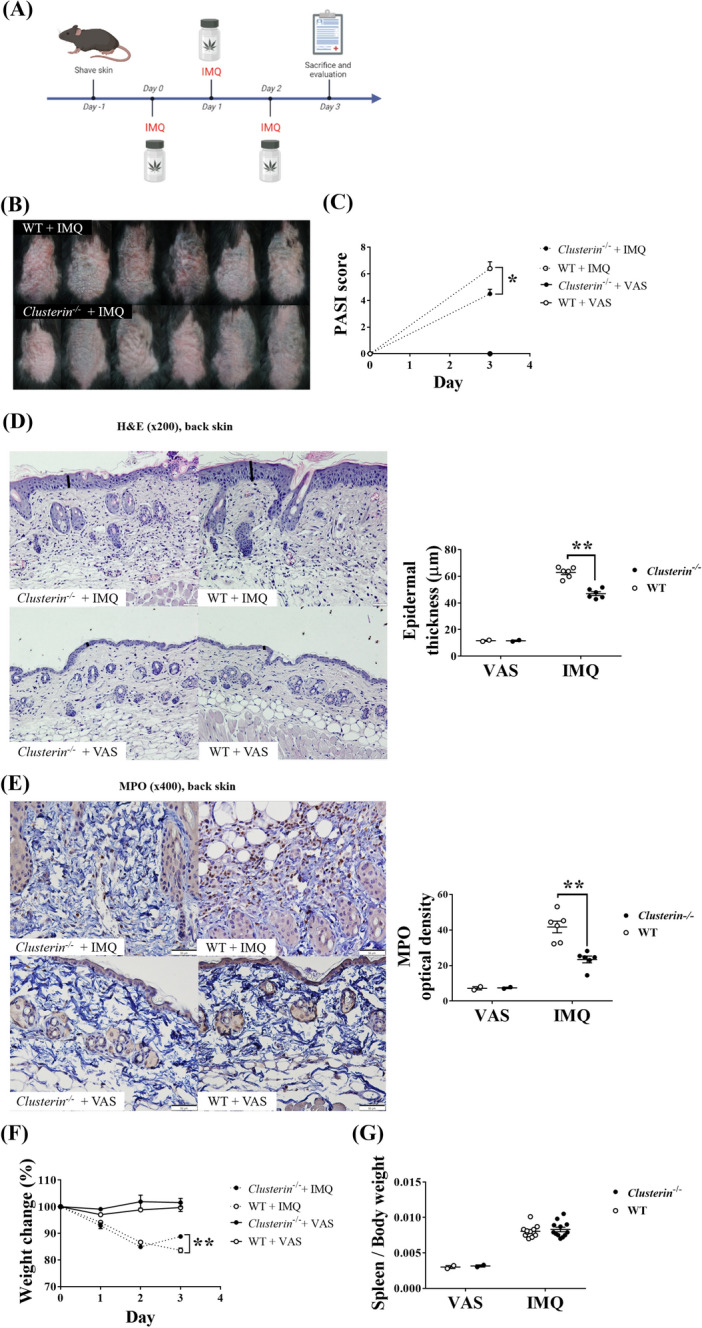
Clinical and histological changes in WT and *clusterin*^−/−^ mice after induction of acute psoriasis-like inflammation by IMQ. (**A**) The experimental scheme of short-term IMQ administration on WT and *clusterin*^−/−^ mice, was generated using Biorender (©BioRender-biorender.com, CA, USA). (**B**) IMQ-treated WT mice (top) showed psoriasis-like changes in the skin, including erythema, scaliness, and increased thickness. However, IMQ-treated *clusterin*^−/−^ mice (bottom) had relatively non-inflamed skin. (**C**) Clinical evaluation of psoriatic skin inflammation using the PASI score. On day 3, WT mice had significantly higher PASI scores than IMQ-treated *clusterin*^−/−^ mice. However, VAS-treated WT and *clusterin*^−/−^ mice scored 0 on day 3. (**D**) H&E staining of IMQ-treated WT and *clusterin*^−/−^ mice. The epidermis of IMQ-treated WT mice was significantly thicker than that of IMQ-treated *clusterin*^−/−^ mice. Magnification: × 200, scale bar: 50 μm. (**E**) Immunohistochemical staining of tissue sections for MPO. The optical density of MPO was significantly higher in IMQ-treated WT mice than in IMQ-treated *clusterin*^−/−^ mice. Magnification: × 400, scale bar: 50 μm. (**F**) During the 3-day treatment with either VAS or IMQ, VAS-treated mice retained their body weight, whereas IMQ-treated mice gradually lost body weight. On day 3, the body weight loss was more significant in IMQ-treated WT mice than in IMQ-treated *clusterin*^−/−^ mice. (G) The ratios of the spleen weight/body weight in IMQ-treated WT and *clusterin*^−/−^ mice was higher than that in VAS-treated WT and *clusterin*^−/−^ mice, with no significant difference between IMQ-treated WT and *clusterin*^−/−^ mice. *P < 0.05, **P < 0.01, ***P < 0.001.

To evaluate the role of clusterin in psoriasis, IMQ was applied to the shaved skin of clusterin-knockout (*clusterin*^−/−^) and WT mice for 3 days (n = 6 per group), while the control group was treated with Vaseline (VAS) (n = 2 per group) for the same 3 days (Fig. 1A). WT mice treated with IMQ showed definite psoriasis-like changes, including increased skin erythema, scaliness, and thickness on the backs and ears, compared to *clusterin*^−/−^ mice treated with IMQ (Fig. 1B). On day 3, the psoriasis area and severity index (PASI) scores for the skin lesions in the IMQ-treated groups were elevated but were 0 in the VAS-treated groups (Fig. 1C). In contrast, the PASI scores of IMQ-treated WT mice were significantly higher than those of IMQ-treated *clusterin*^−/−^ mice (*P* = 0.017). To better characterize the effect of clusterin on psoriasis-like skin inflammatory reactions, we performed histological analyses of dorsal skin sections obtained from *clusterin*^−/−^ and WT mice treated with IMQ or VAS (Fig. 1D). The IMQ-treated mice showed increased epidermal thickness, hyperkeratosis, and epidermal rete-ridge projections into the dermis. The psoriasis-like skin phenotype was less severe in IMQ-treated *clusterin*^−/−^ mice than in IMQ-treated WT mice and was not apparent in VAS-treated mice. The epidermis of IMQ-treated WT mice was significantly thicker than that of IMQ-treated *clusterin*^−/−^ mice (*P* = 0.002). To evaluate neutrophil infiltration into IMQ-induced psoriasis-like skin lesions, we performed immunostaining for myeloperoxidase (MPO) (Fig. 1E). The number of MPO^+^ cells infiltrating the skin lesions of IMQ-treated WT mice was significantly higher than that of *clusterin*^−/−^ mice (*P* = 0.002).

To examine the effect of clusterin on systemic inflammation, we monitored weight changes during the experiment and calculated the spleen weight/body weight ratios (Fig. 1F,G). While both IMQ-treated WT and *clusterin*^−/−^ mice lost weight during the experiment, IMQ-treated WT mice lost significantly more weight than IMQ-treated *clusterin*^−/−^ mice (*P* = 0.002). However, there was no significant difference in relative spleen weight between the two groups (*P* = 0.516).

### Clusterin is associated with development of medium-term IMQ-induced psoriasis-like skin inflammatory reactions in *IL-10*^−/−^ mice

**Figure 2:**
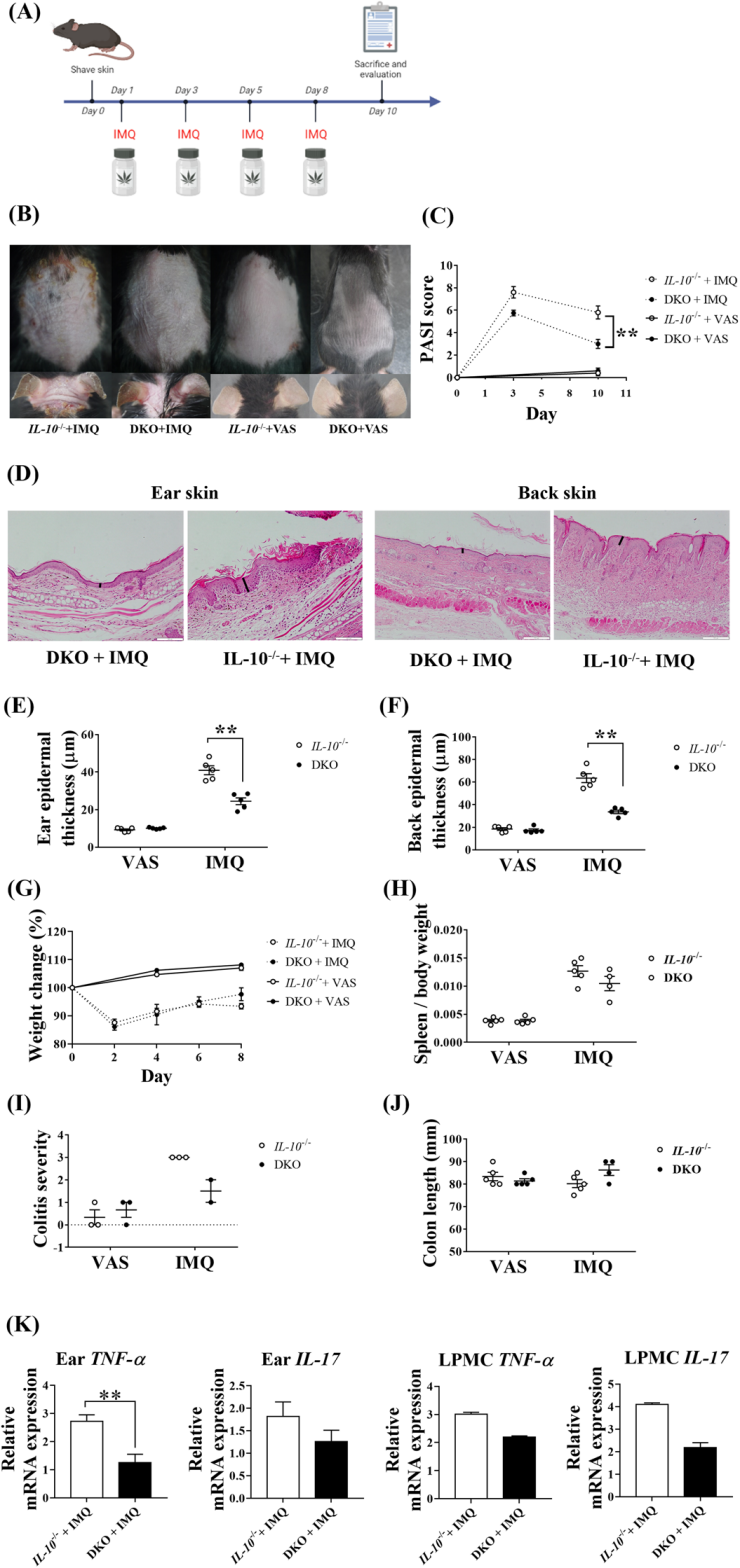
Clinical and histological changes in *IL-10*^−/−^ and DKO mice after medium-term administration of IMQ to induce psoriasis-like inflammation. (**A**) The experimental scheme of IMQ application on *IL-10*^−/−^ and DKO mice, was generated using Biorender. (**B**) IMQ-treated *IL-10*^−/−^ mice showed psoriatic skin changes, while IMQ-treated DKO mice and VAS-treated mice maintained a relatively non-inflamed appearance. (**C**) On day 10, IMQ-treated *IL-10*^−/−^ mice had significantly higher PASI scores than IMQ-treated DKO mice. (**D**) IMQ treatment induced histological psoriatic change, with IMQ-treated *IL-10*^−/−^ mice showing more prominent change than that of IMQ-treated DKO mice. (**E**,**F**) After topical application of IMQ, *IL-10*^−/−^ mice had significantly thicker ear and back epidermis than DKO mice. (**G**) IMQ-treated mice tended to lose weight during the experiment. (**H**) IMQ-treated *IL-10*^−/−^ and DKO mice had heavy spleens, but there was no significant difference in the ratios of the spleen weight/body weight between IMQ-treated *IL-10*^−/−^ and IMQ-treated DKO mice. (**I**,**J**) Colitis severity and colon length were evaluated to assess colitis development after IMQ treatment; there were no significant differences among the groups, but IMQ-treated *IL-10*^−/−^ mice appeared to have more severe colitis than other mice. (**K**) RNA was extracted from ear skin and colon LPMCs, and RT-qPCR was used to determine the expressions of TNF-α and IL-17. The expression of TNF-α was significantly higher in ear tissues from IMQ-treated *IL-10*^−/−^ mice than those from IMQ-treated DKO mice. The expression of TNF-α in LPMCs as well as that of IL-17 in ear tissues and LPMCs were higher in IMQ-treated *IL-10*^−/−^ mice than those in IMQ-treated DKO mice; however, these differences were statistically insignificant. *P < 0.05, **P < 0.01, ***P < 0.001.

Medium-term topical application of IMQ to IL-10-knockout (*IL-10*^−/−^) mice promotes psoriasis-like skin inflammation^6^. To evaluate the effect of clusterin on IMQ-induced psoriasis-like inflammatory reactions in *IL-10*^−/−^ mice, we treated *IL-10*^−/−^ and IL-10/clusterin double knockout (DKO) mice with topical IMQ or VAS on Days 1, 3, 5, and 8 (n = 5 per group) and sacrificed the mice on day 10 (Fig. 2A). After treatment with IMQ, *IL-10*^−/−^ mice showed a psoriasis-like phenotype, whereas DKO mice did not (Fig. 2B). The PASI scores for skin lesions in IMQ-treated *IL-10*^−/−^ and DKO mice increased on day 3, but decreased on day 10 (Fig. 2C). The PASI scores of IMQ-treated *IL-10*^−/−^ mice on day 10 were significantly higher than those of IMQ-treated DKO mice (*P* = 0.008). Hematoxylin and eosin (H&E) staining revealed that skin tissues from IMQ-treated *IL-10*^−/−^ mice showed more prominent psoriatic changes than those from IMQ-treated DKO mice. Specifically, they showed a loss of the granular layer, wafer-like scales with neutrophils, and psoriasiform hyperplasia (Fig. 2D). The thicknesses of the ear and back skin were measured to quantify the severity of psoriasis-like skin inflammation (Fig. 2E,F). Skin specimens from IMQ-treated *IL-10*^−/−^ and DKO mice were thicker than those from both VAS-treated mice. Importantly, the skin of IMQ-treated *IL-10*^−/−^ mice was significantly thicker than that of IMQ-treated DKO mice (*P* = 0.008 for both the ears and back).

To examine the differences in systemic inflammation among the groups, weight reduction during IMQ treatment was monitored and the spleen weight/body weight ratios were calculated. During the topical application of IMQ, both *IL-10*^−/−^ and DKO mice lost weight, which was gradually regained when IMQ treatment stopped (Fig. 2G). In contrast, VAS-treated *IL-10*^−/−^ and DKO mice gained weight throughout the experiment, with no significant differences in weight changes between IMQ-treated *IL-10*^−/−^ and DKO mice. Moreover, the spleen weight/body weight ratios increased in IMQ-treated mice to a greater extent than those in VAS-treated mice, but no significant differences between those of IMQ-treated *IL-10*^−/−^ and DKO mice were observed (Fig. 2H). Next, we assessed the colitis severity and colon length to evaluate the differences in colitis among the groups. Colitis was more severe in IMQ-treated mice than in VAS-treated mice. IMQ-treated *IL-10*^−/−^ mice showed more prominent colitis than IMQ-treated DKO mice, although there was no significant difference between the two groups (Fig. 2I). Although IMQ-treated *IL-10*^−/−^ mice appeared to have the shortest colons, there were no significant differences in colon length between the groups (Fig. 2J). We obtained ear skin and colon lamina propria mononuclear cells (LPMCs) and evaluated their mRNA expression levels (Fig. 2K). The expression of TNF-α mRNA in ear skin from IMQ-treated *IL-10*^−/−^ mice was significantly higher than that in IMQ-treated DKO mice (*P* = 0.005). The expressions of IL-17 mRNA in ear skin or colon LPMCs and of TNF-α mRNA in colon LPMCs in IMQ-treated *IL-10*^−/−^ mice were higher than those in IMQ-treated DKO mice; however, no significant differences were observed.

### Clusterin deficiency alleviates systemic inflammation and colitis in mice with long-term IMQ-induced psoriasis-like skin inflammation

**Figure 3:**
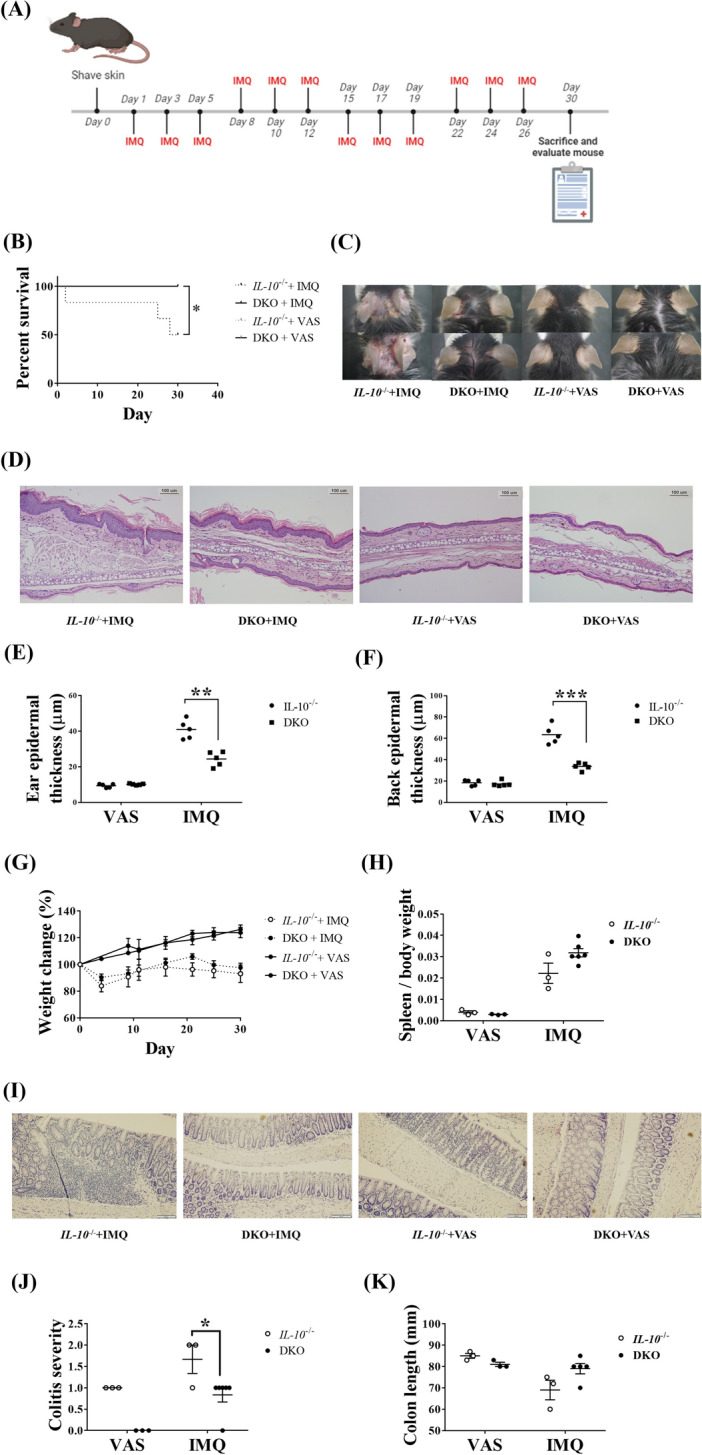
Clinical and histological changes in *IL-10*^−/−^ and DKO mice after long-term application of IMQ to induce psoriasis-like inflammation. (**A**) The experimental scheme of long-term IMQ administration, was generated using Biorender. (**B**) After IMQ application to the ear and shaved back skin for 4 weeks, IMQ-treated DKO mice showed significantly higher survival rates than IMQ-treated *IL-10*^−/−^ mice. (**C**) Long-term IMQ treatment induced psoriatic skin changes that could be alleviated by the genetic deletion of clusterin. (**D**) Representative images of H&E-stained skin sections after 4 weeks of treatment with IMQ. H&E-stained ear and back skin sections from IMQ-treated *IL-10*^−/−^ mice showed more prominent psoriatic skin changes than those of IMQ-treated DKO mice. (**E**,**F**) IMQ-treated *IL-10*^−/−^ mice had a significantly thicker epidermis than IMQ-treated DKO mice. (**G**) IMQ-treated mice failed to gain weight during the 4-week treatment with IMQ. (**H**) The ratios of the spleen weight/body weight in IMQ-treated mice were higher than those in VAS-treated mice. (**I**) Representative images of H&E-stained colon tissues after long-term treatment with IMQ or VAS. IMQ-treated *IL-10*^−/−^ mice showed the most prominent colitis compared to other groups. Magnification: × 200, scale bar: 100 μm. (**J**) After long-term treatment with IMQ, colitis in IMQ-treated *IL-10*^−/−^ mice was significantly more severe than that in IMQ-treated DKO mice. (**K**) IMQ-treated *IL-10*^−/−^ mice tended to have shorter colon than IMQ-treated DKO mice; however, these differences were statistically insignificant. *P < 0.05, **P < 0.01, ***P < 0.001.

Long-term administration of IMQ can reflect systemic inflammatory conditions and comorbidities associated with psoriasis, such as colitis^6^. First, we applied IMQ to the ear and shaved back skin of DKO and *IL-10*^−/−^ mice for a period of 4 weeks (IMQ-treated group, n = 6; VAS-treated group, n = 3). The skin of mice was shaved on day 0, and IMQ or VAS was applied to the skin once every 3 days per week for 4 weeks (Fig. 3A). A 50% survival rate was observed in IMQ-treated *IL-10*^−/−^ mice, whereas all IMQ-treated *DKO* mice survived (Fig. 3B). The survival rate of IMQ-treated *IL-10*^−/−^ mice was significantly lower than that of the mice in the other groups (*P* = 0.042). These findings suggest that in *IL-10*^−/−^ mice, long-term IMQ administration can provoke fatal systemic inflammation, including colitis, while clusterin deficiency can protect mice from this lethal outcome.

Next, IMQ was applied to the ear skin only to induce less severe systemic inflammation, thereby increasing the survival rate of IMQ-treated mice. After a 4-week IMQ treatment period, IMQ-treated *IL-10*^−/−^ mice had more severe psoriatic skin changes than those seen in IMQ-treated DKO mice, while no skin inflammation was observed in VAS-treated mice (Fig. 3C). Skin sections from IMQ-treated *IL-10*^−/−^ mice showed histological characteristics of psoriasis, including increased epidermal thickness, acanthosis, and parakeratosis in the stratum corneum, all of which were much more prominent than those observed in the skin sections of IMQ-treated DKO mice (Fig. 3D). The epidermal layers of the ear and back skin of IMQ-treated *IL-10*^−/−^ mice were significantly thicker than those of IMQ-treated DKO mice (*P* = 0.004, for ear skin; *P* < 0.001, for back skin) (Fig. 3E,F). Moreover, the IMQ-treated mice failed to gain weight during the experiments and had heavier spleens than the VAS-treated mice. However, there were no significant differences in the body weight changes or spleen weight/body weight ratios between the treatment groups (Fig. 3G,H). Murine colon tissues were obtained and H&E staining was performed to evaluate colitis. Mucosal infiltration of inflammatory cells was observed in colon sections from IMQ-treated *IL-10*^−/−^ mice. Scattered inflammatory cells with altered mucosal architecture were observed in the colonic mucosa of the IMQ-treated DKO mice. Mild epithelial hyperplasia and goblet cell loss were observed in the colonic mucosa of the VAS-treated *IL-10*^−/−^ mice. In contrast, colon sections from VAS-treated DKO mice exhibited normal structures without inflammatory cell infiltration (Fig. 3I). IMQ-treated *IL-10*^−/−^ mice had significantly higher colitis scores than IMQ-treated DKO mice (*P* = 0.038) (Fig. 3J). Additionally, the colons of IMQ-treated DKO mice were longer than those of IMQ-treated *IL-10*^−/−^ mice, yet the difference was not statistically significant (Fig. 3K). In summary, clusterin deficiency relieved psoriatic skin inflammation and colitis induced by long-term topical administration of IMQ.

### Suppression of clusterin inhibits the activation of keratinocytes via the Th17 pathway

**Figure 4:**
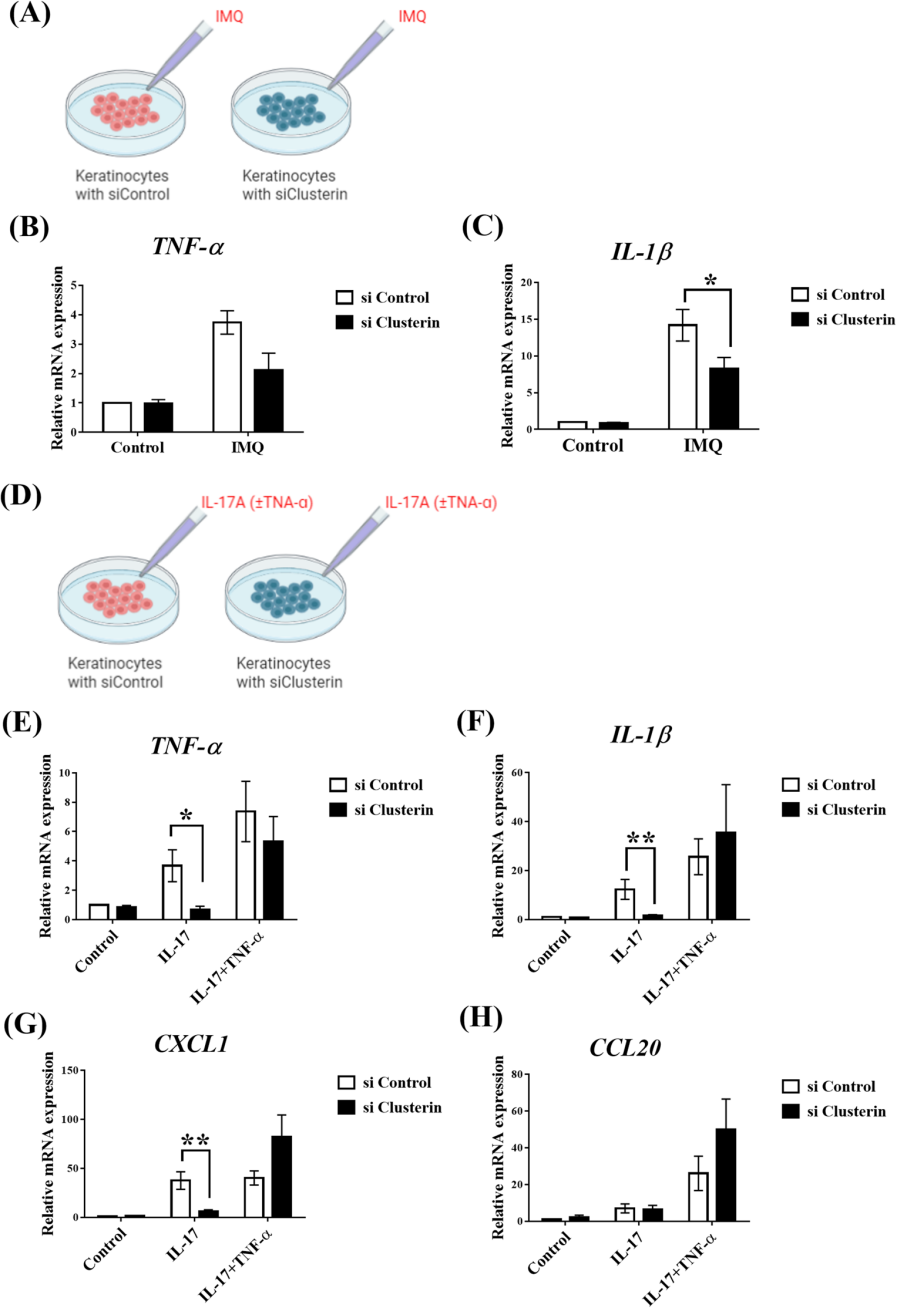
Suppression of clusterin transcription alleviates IMQ and IL17-induced inflammation. (**A**) To elucidate the detailed role of clusterin in IMQ-induced skin inflammation, keratinocytes were transfected with clusterin or control siRNA (siClusterin and siControl, respectively) and stimulated with IMQ. The expression levels of (**B**) *TNF-α* and (**C**) *IL-1β* increased after IMQ treatment, and this was suppressed by silencing clusterin. In particular, the expression of *IL-1β* increased significantly in siControl-treated keratinocytes compared to that in siClusterin-treated keratinocytes. (**D**) Next, siControl- or siClusterin-transfected keratinocytes were stimulated with either IL-17 or both IL-17 and TNF-α. The expression of (**E**) *TNF-α*, (**F**) *IL-1β*, (**G**) *CXCL1*, and (**H**) *CCL20* was evaluated under conditions of IL-17 stimulation or IL-17/TNF-α co-stimulation. When only IL-17 was administrated, the expression of *TNF-α, IL-1β*, and *CXCL1* was significantly higher in keratinocytes transfected with siControl than in keratinocytes transfected with siClusterin. However, upon co-stimulation with IL-17/TNF-α, there were no significant differences in inflammatory cytokine expressions. *P < 0.05, **P < 0.01, ***P < 0.001.

The central role of the IL-23/IL-17 axis in psoriasis has been demonstrated previously^2^; however, keratinocytes are also involved in this process. When exposed to multiple triggers such as IMQ, stressed keratinocytes produce inflammatory cytokines, including IL-1β, IL-6, and TNF-α, which contribute to dermal DC activation and the initiation of psoriasis. Subsequently, Th17 cells mature via dermal DCs and migrate to the skin, where they produce IL-17. IL-17 stimulates keratinocytes leading to neutrophil recruitment and epidermal hyperplasia, which contributes to psoriasis plaque progression. Clusterin is produced in human keratinocytes and its levels increase under stress conditions^28,29^. Therefore, to examine the involvement of clusterin in the initiation of psoriasis, human keratinocytes transfected with clusterin or control small interfering RNA (siRNA) were cultured and stimulated with IMQ (Fig. 4A). There was no significant attenuation in the expressions of TNF-α and IL-1β with clusterin siRNA in the absence of IMQ (control) (Fig. 4B,C). However, the expression of TNF-α was increased in cells treated with IMQ, while clusterin silencing suppressed the expression of TNF-α, although the difference was not statistically significant. Furthermore, clusterin siRNA significantly suppressed the expression of IL-1β in the presence of IMQ compared to control siRNA (*P* = 0.048).

To mimic the molecular pathways involved in psoriatic plaque progression, keratinocytes were transfected with clusterin or control siRNA and stimulated with IL-17 and TNF-α (Fig. 4D). Clusterin siRNA significantly inhibited the increase in the expression of TNF-α (*P* = 0.032), IL-1β (*P* = 0.008), and CXCL1 (*P* = 0.008) in human keratinocytes stimulated with IL-17 (Fig. 4E–G). However, the expression of CCL20 was not significantly different with or without clusterin knockdown (Fig. 4H). Moreover, when human keratinocytes were stimulated with both TNF-α and IL-17, clusterin siRNA failed to inhibit the upregulated expression of TNF-α, IL-1β, CXCL1, and CCL20. Taken together, these results suggest that clusterin suppresses the expression of pro-inflammatory cytokines involved in the signaling pathways of both psoriasis initiation and plaque progression.

### Gut microbiome of IMQ-treated *clusterin*^−*/*−^ mice differs from that of IMQ-treated WT mice

**Figure 5:**
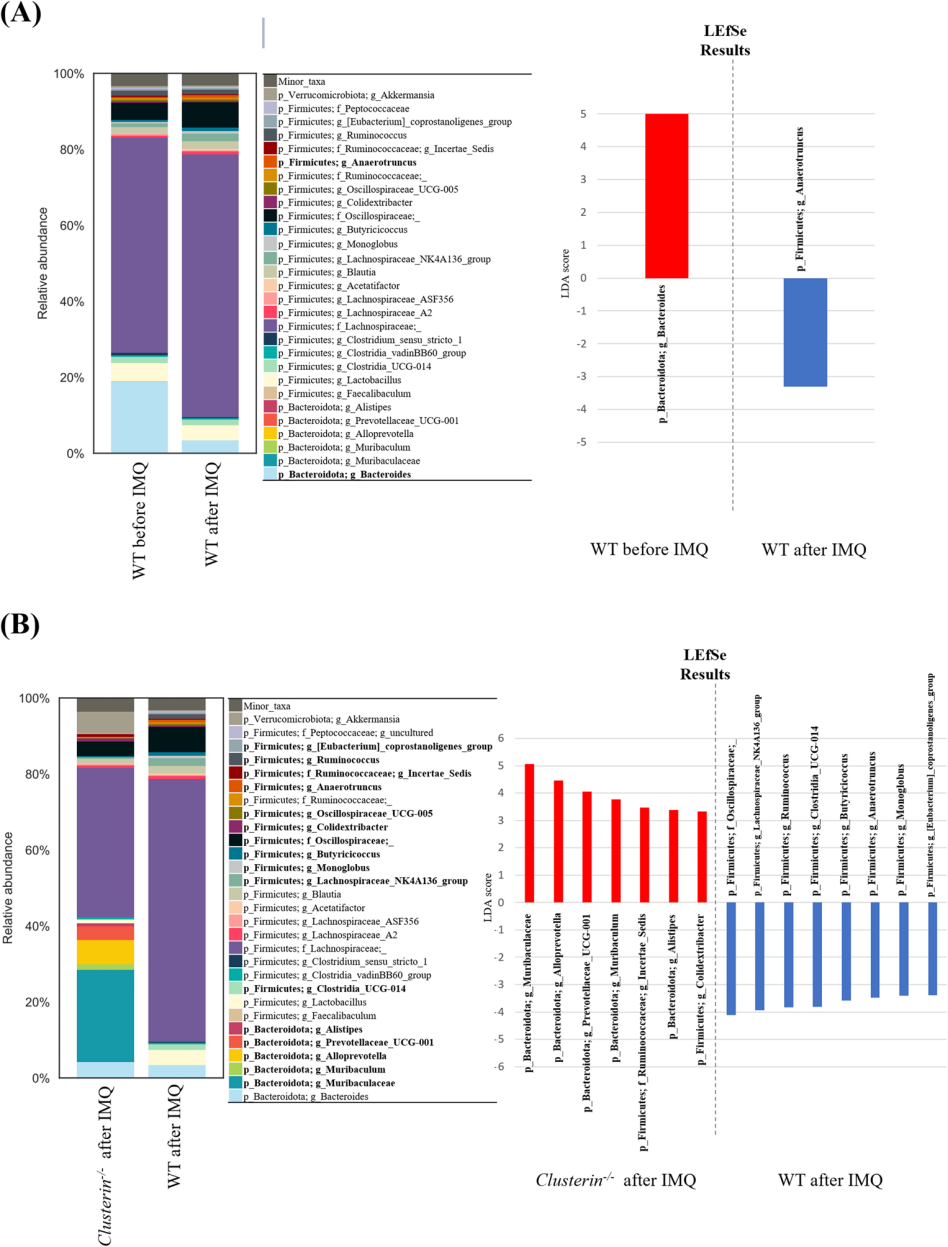
Genetic deletion of clusterin alters the intestinal microbiota. (**A**) Relative abundance and LDA scores of the differential abundant genera taxa are displayed. Taxa enriched in the intestinal microbiota of mice before IMQ treatment (red) or after IMQ treatment (blue) are indicated by a positive or negative LDA score, respectively. There was a decrease in the abundance of *Bacteroides* and an increase in that of *Anaerotruncus* after IMQ treatment. (**B**) Taxa enriched in the intestinal microbiota of IMQ-treated *clusterin*^−/−^ mice (red) or IMQ-treated WT mice (blue) are indicated by a positive or negative LDA score, respectively. Briefly, notable increases in bacteria from the Bacteroidota phylum and reductions of bacteria the Firmicutes phylum were detected in IMQ-treated *clusterin*^−/−^ mice compared to IMQ-treated WT mice. *P < 0.05, **P < 0.01, ***P < 0.001.

Given that alterations in the intestinal microbiota can affect host inflammation at local and systemic levels^30^, the regulation of psoriasis and colitis by clusterin may be associated with intestinal dysbiosis. To investigate the effects of genetic deletion of clusterin on gut bacterial communities, fecal samples were collected from WT and *clusterin*^−*/*−^ mice prior to topical administration of IMQ and again after 3 days of treatment with IMQ. Subsequently, the bacterial composition was examined by 16S rRNA gene sequencing (n = 6, WT; n = 5, *clusterin*^−*/*−^). The results showed no significant differences in the Shannon index (*P* = 0.078) or Faith phylogenetic diversity index (*P* = 0.262) of WT mice before and after IMQ treatment (Fig. S1A and S1B). β-diversity analyses performed on unweighted and weighted UniFrac distance matrices and PCoA revealed significant clustering (*P* = 0.028, weighted UniFrac distance; *P* = 0.006, unweighted UniFrac distance) of data from WT mice before and after IMQ treatment (Fig. S1C and S1D). Linear discriminant analysis (LDA) effect size (LEfSe) was used to identify the key bacteria related to psoriatic skin inflammation. At the phylum level, the relative abundance of Bacteroidota decreased and that of Firmicutes increased after WT mice received topical IMQ. At the genus level, *Bacteroides* (LDA = 5.027, *P* = 0.016) was more abundant in WT mice before IMQ treatment than after IMQ treatment, and a significantly higher abundance of *Anaerotruncus* (LDA = 3.303, *P* = 0.016) was observed in WT mice after IMQ treatment than before IMQ treatment (Fig. 5A).

The α-diversity results revealed significant differences in the gut microbiota composition of WT and *clusterin*^−*/*−^ mice after IMQ treatment, as shown by differences in the Shannon index (*P* = 0.011). However, there were no significant differences in Faith’s phylogenetic diversity index (*P* = 0.583) (Fig. S2A and S2B). Additionally, we found a significant difference in the diversity of the gut microbiome, as demonstrated by the weighted and unweighted UniFrac distance matrices according to clusterin expression (*P* = 0.002, weighted UniFrac distance; *P* = 0.003, unweighted UniFrac distance) (Fig. S2C and S2D). LEfSe identified 10 bacterial genera enriched in IMQ-treated *clusterin*^−*/*−^ mice and 11 bacterial genera enriched in IMQ-treated WT mice (Fig. 5B). Among these genera, *Muribaculaceae* (LDA = 5.064, *P* = 0.006), *Alloprevotella* (LDA = 4.460. *P* = 0.006), *Prevotellaceae* (LDA = 4.051, *P* = 0.006), *Muribaculum* (LDA = 3.770, *P* = 0.005), *Incertae sedis* (LDA = 3.469, *P* = 0.006), *Alistipes* (LDA = 3.390, *P* = 0.006), and *Colidextribacter* (LDA = 3.338, *P* = 0.018) were significantly more abundant in IMQ-treated *clusterin*^−*/*−^ mice than in IMQ-treated WT mice. However, *Oscillospiraceae* (LDA = 4.117, *P* = 0.028), *Lachnospiraceae* (LDA = 3.935, *P* = 0.018)*, Ruminococcus* (LDA = 3.822, *P* = 0.006)*, Clostridia* (LDA = 3.811, *P* = 0.006)*, Butyricicoccus* (LDA = 3.586, *P* = 0.006)*, Anaerotruncus* (LDA = 3.485, *P* = 0.006)*, Monoglobus* (LDA = 3.411, *P* = 0.004), and *Eubacterium* (LDA = 3.395, *P* = 0.004) were significantly more abundant in IMQ-treated WT mice than in IMQ-treated *clusterin*^−*/*−^ mice. Thus, changes in the gut bacterial composition could explain the less severe skin inflammation observed in *clusterin*^−*/*−^ mice following topical application of IMQ.

### Clusterin has no significant effect on intestinal permeability in the murine psoriatic inflammation model

To evaluate the effect of clusterin on intestinal barrier function, we measured intestinal permeability in IMQ-treated WT and *clusterin*^−*/*−^ mice using FITC-dextran (Supplementary Material). Following topical administration of IMQ, WT mice showed greater intestinal permeability than before topical administration of IMQ (*P* = 0.010 at 5 min; *P* = 0.016 at 15 min; *P* = 0.029 at 30 min; *P* = 0.029 at 60 min) (Fig. S3A). However, there were no significant differences in intestinal permeability between the IMQ-treated WT and *clusterin*^−*/*−^ mice (*P* = 0.095 at 5 min; *P* = 0.841 at 15 min; *P* = 0.400 at 30 min; *P* = 0.200 at 60 min) (Fig. S3B). These data suggest that increased intestinal permeability could explain the more severe colitis observed in the IMQ-induced psoriatic inflammation model; however, this increase in intestinal permeability does not appear to be linked to clusterin.

### Patients with psoriasis show higher expression of clusterin than controls

**Figure 6:**
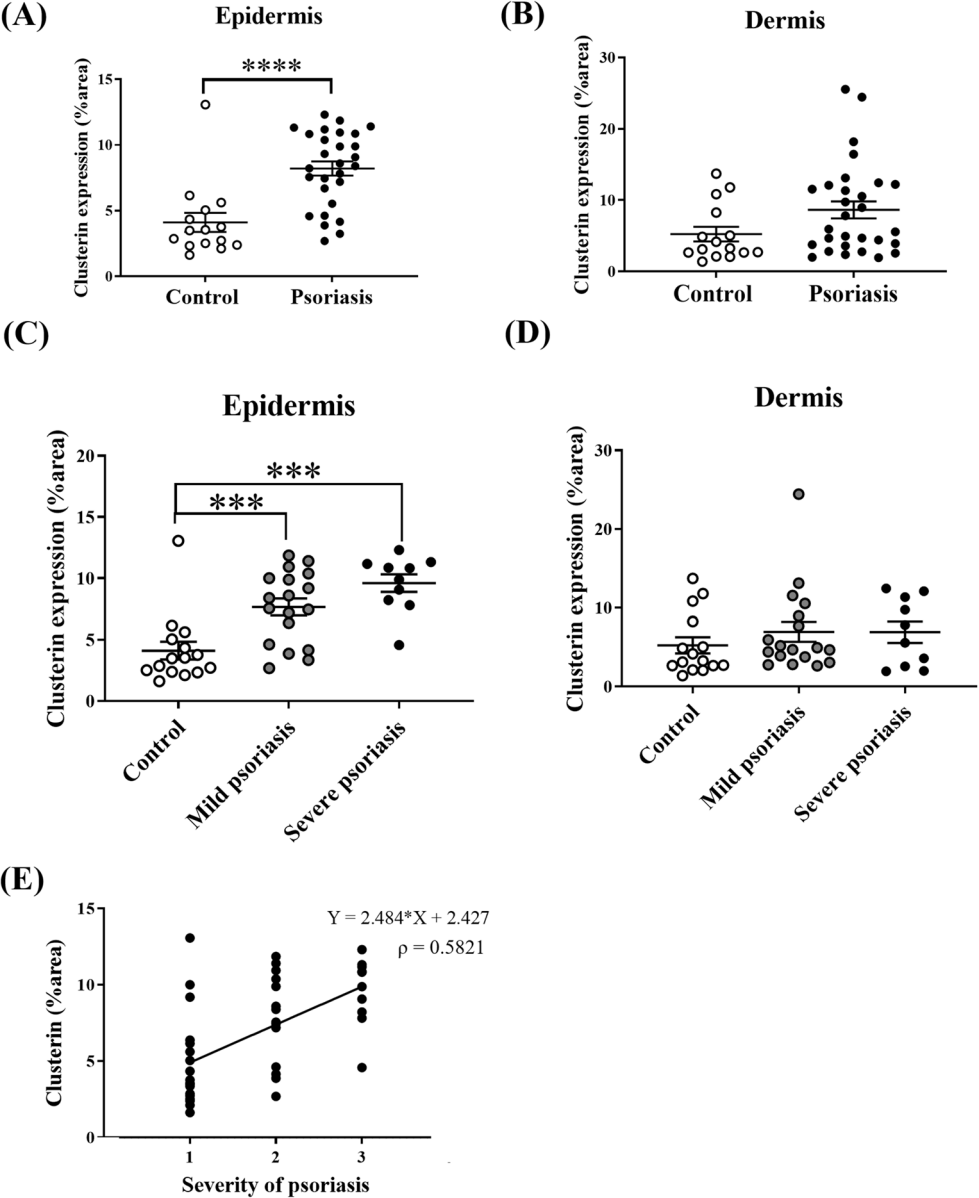
Clusterin expression in the epidermis of patients with psoriasis is higher than that in healthy controls. (**A**–**D**) Skin samples were obtained from patients with psoriasis and from healthy controls, and immunohistochemical analysis of clusterin expression was performed. Clusterin-positive areas (%) in the epidermis were significantly larger in patients with psoriasis than in controls. (**E**) Scatter plot and Spearman’s correlation analysis revealed that psoriasis severity correlated positively with clusterin expression. *P < 0.05, **P < 0.01, ***P < 0.001.

Skin samples were obtained from patients with psoriasis and healthy controls and subjected to immunohistochemical analyses to evaluate the differences in clusterin expression between the psoriasis and control groups (psoriasis group, n = 29; control group, n = 15). Clusterin expression was higher in the skin samples from patients with psoriasis than in those from healthy controls (Fig. S4). The mean clusterin-positive areas (% ± SEM) in the epidermis of the psoriasis and control groups were 8.21% ± 0.54% and 4.11% ± 0.73%, respectively. The clusterin-positive area in the epidermis of the psoriasis group was significantly larger than that in the control group (*P* < 0.001; 8.63% ± 1.19% vs. 5.25% ± 1.02%, respectively) (Fig. 6A), and there were no significant differences in the size of clusterin-positive areas in the dermis between the psoriasis and control groups (*P* = 0.067) (Fig. 6B).

Clusterin expression was examined according to the severity of psoriasis, using skin samples from patients with psoriasis and healthy controls. The mean clusterin-positive area (% ± standard error of the mean [SEM]) in the epidermis was 7.66% ± 0.69% in the mild psoriasis group and 9.61% ± 0.72% in the severe psoriasis group. Clusterin expression in the epidermis was significantly higher in the mild and severe psoriasis groups than in the control group (*P* < 0.001, control vs*.* mild psoriasis; *P* < 0.001, control vs. severe psoriasis) (Fig. 6C). The clusterin-positive area in the dermis of the mild psoriasis group was 6.95% ± 1.27%, while that in the severe psoriasis group was 6.91% ± 1.36%. Additionally, there were no significant differences in clusterin expression in the dermis of patients with mild psoriasis, severe psoriasis, and no psoriasis (*P* = 0.178, control vs. mild psoriasis; *P* = 0.495, control vs*.* severe psoriasis; *P* = 0.981, mild psoriasis vs. severe psoriasis) (Fig. 6D). Moreover, clusterin expression was significantly correlated with psoriasis severity (*P* < 0.001, Spearman’s rho = 0.5821) (Fig. 6E).

## Discussion

We demonstrated that the genetic deletion of clusterin relieved psoriasis-like skin inflammation, systemic inflammation, and colitis in short-, medium-, and long-term IMQ-induced murine psoriasis model. Topical administration of IMQ induces transient psoriasis-like skin inflammation by modulating the IL-23/IL-17 axis^4^. In IMQ-induced chronic psoriasis, *IL-10*^−/−^ mice exhibited more severe skin and systemic inflammation than WT mice^6^. WT mice recovered well during the intervals between IMQ treatments, which was not suitable as an experimental model of chronic inflammatory reactions of psoriasis; however, psoriatic skin inflammation was maintained in IMQ-treated *IL-10*^−/−^ mice. Moreover, when IMQ was applied to both ears and shaved back skin in the long-term IMQ-induced chronic psoriasis model, approximately 50% of the IMQ-treated *IL-10*^−*/*−^ mice failed to survive (Fig. 3B). Therefore, IMQ was applied only to the ears in the subsequent experiments. Following the application of IMQ to the ear only, all mice survived the 4-week experimental period, following which IMQ-treated *IL-10*^−*/*−^ mice had significantly more severe colitis than IMQ-treated DKO mice (Fig. 3J). These results indicate that if psoriatic skin inflammation is induced to a sufficiently large extent, systemic inflammation may be life-threatening. Topical IMQ treatment can induce systemic inflammation through elevating serum levels of IL-17A and TNF-α^6^, and increase blood glucose levels, muscle triglyceride levels, and pancreatic β-cell insulin secretion^31^.

Upon stimulating human keratinocytes with IMQ, siRNA-induced clusterin suppression alleviated the expression of pro-inflammatory cytokines. Clusterin has both anti- and pro-inflammatory effects. Studies have shown that clusterin is associated with pro-inflammatory reactions in inflammatory myopathy, obesity, and asthma^18,32,33^, but exerts an anti-inflammatory function in renal injury and autoimmune myocarditis^34,35^. Here, we found that clusterin facilitates systemic inflammation and colitis, indicating that its effects are not limited to local skin inflammation. Moreover, clusterin levels are increased in the skin tissues of patients with psoriasis. Additionally, clusterin levels in the epidermis of patients with psoriasis were higher than those in healthy controls, a phenomenon that correlates with psoriasis severity.

Production of pro-inflammatory cytokines by human keratinocytes was suppressed by transfection with clusterin siRNA despite stimulation by IL-17. Clusterin appears to be a promising therapeutic target, as it can attenuate not only the molecular process involved in psoriasis initiation, but also that of psoriatic plaque progression via IL-17. However, the anti-inflammatory effects of clusterin siRNA were insufficient to invalidate the synergistic action of IL-17 and TNF-α^36^. Studies on the relationship between clusterin and the IL-23/IL-17 axis are lacking; however, clusterin expression shows a positive relationship with IL-17 production in a murine model of Alzheimer’s disease^37^. IL-17 and TNF-α are key players in inducing and maintaining psoriatic inflammation, but stimulate the expression of different genes in keratinocytes^36^. Anti-IL-17 agents show impressive clinical, histological, and transcriptional resolution of psoriasis, while anti-TNF-α agents can induce paradoxical psoriasis in patients with autoimmune diseases other than psoriasis^38,39^. Thus, clusterin could be related to the IL-17 inflammatory pathway, and clusterin antagonists could be good candidates for new therapeutic agents for psoriasis by blocking the IL-17 inflammatory pathway.

Our results suggest that intestinal dysbiosis occurs in IMQ-treated WT mice and is characterized by a shift in the β-diversity and alterations in the relative abundances of certain bacterial taxa. The microbiota composition of IMQ-treated WT mice at the phylum level was characterized by a reduction in Bacteroidota and an increase in Firmicutes; this resulted in an increase in the Firmicutes/Bacteroidota (F/B) ratio in IMQ-treated WT mice compared to that in IMQ-naïve WT mice. Firmicutes and Bacteroidota constitute more than 90% of the intestinal bacterial population. The F/B ratio is related to intestinal homeostasis, and changes in this ratio indicate intestinal dysbiosis. Previous studies have shown that the F/B ratio is higher in patients with psoriasis than in healthy controls^40–43^. Specifically, the F/B ratio is positively correlated with the severity of psoriasis^44^. Furthermore, an increase in the F/B ratio is associated with obesity. A high F/B ratio has also been observed in hypertensive rat models (i.e., spontaneously hypertensive and chronic angiotensin II infusion rat models) and familial hypocholesterolemia mouse models (*Ldlr*^−/−^)^45,46^. Similarly, the F/B ratio is high in patients with type 2 diabetes mellitus (DM) and alterations in this ratio are associated with the severity of type 2 DM^47,48^. As patients with psoriasis have an increased risk of cardiovascular diseases such as hypertension, dyslipidemia, type 2 DM, and obesity^49^, intestinal dysbiosis indicated by an increased F/B ratio may explain this association. However, it remains unclear whether intestinal dysbiosis is the cause or consequence of these disorders^50^. At the genus level, we found a reduction in *Bacteroides* and an increase in *Anaerotruncus* in IMQ-treated WT mice compared to IMQ-naïve WT mice. Other studies investigating patients with psoriasis have also reported a reduction in *Bacteroides*^43,51^.

Interestingly, an increase in bacteria belonging to the phylum Bacteroidota and a decrease in most bacteria belonging to the phylum Firmicutes were found in IMQ-treated *clusterin*^−*/*−^ mice compared to IMQ-treated WT mice. However, our data also showed that the increased intestinal permeability after topical administration of IMQ did not recover through the genetic deletion of clusterin. Recently, the importance of the “gut–skin axis” in the pathogenesis of psoriasis has been highlighted in research on both patients with psoriasis and psoriasis animal models^52–56^. For example, bacterial DNA translocation into the blood may result from increased intestinal permeability in patients with active psoriasis, thereby potentially triggering systemic inflammation^57^. Furthermore, recovery from small intestinal bacterial overgrowth may attenuate the erythema of psoriasis lesions in patients^56^. Similarly, mice treated with broad-spectrum antibiotics demonstrate milder IMQ-induced psoriasiform lesions with reduced local and systemic Th17 activation. Although there are no studies on the relationship between intestinal dysbiosis and serum clusterin levels in patients with psoriasis, the changes in gut microbiome components and metabolites in patients with Alzheimer’s disease are associated with changes in the serum clusterin level^37^. These findings support the importance of the intestinal microbiota in the pathogenesis of psoriasis. In the present study, genetic deletion of clusterin arrested or reversed the increase in the F/B ratio but did not normalize the increased intestinal permeability. Further studies are required to investigate how clusterin and the intestinal microbiota interact to regulate systemic inflammation.

Collectively, our results suggest that clusterin modulates psoriasiform skin lesions, systemic inflammation (including colitis), and intestinal dysbiosis in an animal model of IMQ-induced psoriasis. These findings are also consistent with the results of human studies, as clusterin expression has been shown to be increased in the epidermis of patients with psoriasis in a severity-dependent manner. Also, siRNA-induced clusterin suppression alleviated the production of pro-inflammatory cytokines by human keratinocytes, which are important for the initiation and progression of psoriasis. These data suggest that clusterin may be a useful marker of psoriasis severity and a potential therapeutic target for localized psoriatic skin inflammation and associated comorbidities. However, the specific mechanism by which clusterin regulates the “gut–skin axis” remains unclear, as does the role of clusterin in human psoriasis comorbidities. Additionally, as current treatments associated with the “gut–skin axis,” such as probiotics and fecal microorganism transplantation, have demonstrated heterogenous effects on psoriasis^58,59^, further studies are required.

## Materials and methods

### Mice

WT C57/BL6 mice were purchased from Orient (Seongnam, Korea), *IL-10*^−/−^ mice were supplied by the Center for Animal Resource and Development (Seoul, Korea), and *clusterin*^−/−^ mice were kindly donated by Professor M.S. Kim (Ulsan University College of Medicine, Seoul, Korea). Homozygous *IL-10*^−/−^ and *clusterin*^−/−^ mice with a C57/BL6 background were pair-bred, and heterozygous DKO mice were identified by genotyping. All mouse experiments were approved by the Institutional Animal Care and Use Committee of the SMG-SNU Boramae Medical Center (Institutional Review Board [IRB] No. 2020-0046, 2020-0052) were performed in accordance with the relevant guidelines, regulations, and ARRIVE guidelines. All mice were maintained in the Laboratory of Experimental Animal Research of the SMG-SNU Boramae Medical Center under specific pathogen-free conditions with a standard temperature, humidity, and light/dark cycle. Mice with a weight reduction of more than 20% from baseline were excluded from the experiments.

### IMQ-induced psoriasis-like inflammatory reactions in mice

Seven- to eight-week-old *clusterin*^−/−^ and WT mice were randomly divided into two groups, namely IMQ and VAS groups, for acute IMQ-induced psoriasis-like inflammation models. IMQ-induced psoriasis-like inflammation was induced by topical administration (on 3 consecutive days) of IMQ cream (5% Aldara^®^; 3 M Pharmaceuticals, MN, USA) to the ear or shaved back skin of the mice. VAS cream was applied to the skin of control mice. Because IL-10 deficiency provokes prominent systemic inflammation^6^, 7–8-week-old DKO and *IL-10*^−/−^ mice were used as models of medium- and long-term IMQ-induced psoriasis-like inflammation. IMQ was applied to the ear or shaved back skin of the DKO and *IL-10*^−/−^ mice three times a week for 4 weeks. The investigators could not be blinded to whether the mice were treated with IMQ or VAS because of the differences in overt skin inflammation.

The PASI score, epidermal thickness (μm), MPO optical density, weight change (%), spleen weight/body weight ratio, colitis severity, and colon length (mm) were assessed. Body weight was measured daily in the acute experimental model and every other day in the chronic experimental model. The weight change was calculated as follows: weight change (%) = [(subsequent weight) – (initial weight)] × 100. The mice were euthanized by isoflurane inhalation, and the skin, colon, and spleen samples were obtained. The colon length and spleen weight were measured.

### Assessment of the severity of skin inflammation

Gross skin inflammation in the murine experimental model was evaluated using the PASI score. The PASI score was calculated as the sum of three indices: erythema, thickness, and scale score. Erythema, thickness, and scale scores were independently scored from 0 to 4, where the more severe the erythema, thickness, or scale, the higher the PASI score.

### Histological and immunohistochemical analyses

The skin samples were collected, fixed in 4% paraformaldehyde and embedded in paraffin. The skin sections were stained with H&E and an antibody specific for MPO (1:25; Abcam, Cambridge, UK). Epidermal thickness (× 200, average of two random measurements) and optical density of MPO (× 400, average of two random measurements) were analyzed using ImageJ software (National Institutes of Health, Bethesda, MD, USA).

### Colitis severity

The colons were collected, fixed in 4% buffered formalin for 24 h, and embedded in paraffin. Colon tissues were sectioned and stained with H&E. Histological assessment was scored on a scale of 0–4 as follows: 0, no significant lesions; 1, minimal to mild inflammatory infiltrates in the lamina propria or submucosa; 2, mild to moderate inflammatory infiltrates, occasional edema, and limited focal ulceration; 3, moderate to marked inflammation, edema, and necrosis of up to 70% of the mucosa; and 4, marked transmural inflammation, edema, and necrosis affecting 70–100% of the mucosa.

### Culture and preparation of human keratinocytes

HaCaT cells (human keratinocyte cells) were provided by Professor J.H. Chung (Department of Dermatology, Seoul National University College of Medicine, Seoul, Korea) and originated from skin tissues obtained from a patient who had circumcision surgery. HaCaT cells were cultured in Dulbecco’s modified Eagle’s medium/Earle’s balanced salt solution medium supplemented with 10% fetal bovine serum, 1 mM non-essential amino acids, 1 mM sodium pyruvate, and 2 mM sodium bicarbonate without antibiotics. All cells were incubated at 37 °C in 5% CO_2_. Cells were transfected with siRNA at 60%–80% cell confluency. Briefly, clusterin or control siRNAs were diluted in a serum–free medium and mixed with a transfection reagent (WelGene, Gyeongsan-si, Korea). After 10 min of incubation, the mixture was added to keratinocytes. After transfection, cells were treated with IMQ cream or cytokines.

A total of 250 mg of 5% IMQ cream or vehicle was dissolved in 1.5 mL dimethylsulphoxide and 2 μL of the mixture was added to 200 μL of Opti-MEM (Thermo Fischer Scientific, MA, USA), followed by addition of 6 μL of Lipofectamine 3000 (Thermo Fischer Scientific). After 20 min, the mixture was added to the keratinocytes for 16 h before harvesting. After siRNA transfection, the medium was supplemented with or without IL-17 (200 ng/mL; R&D Systems, MN, USA) and TNF-α (10 ng/mL; Sigma-Aldrich Corp, MO, USA) for 24 h before harvesting.

### Quantitative reverse transcription PCR (RT-qPCR)

Total mRNA was extracted from murine ear skin and colon LPMCs collected from the colon, as previously described^60^. Briefly, after the cecum was removed, the colon was cut into small pieces (< 0.5 cm) and cleaned. Tissues were incubated at 37 °C for 20 min with 1 mM dithiothreitol and the supernatant was removed using nylon mesh. The tissues were incubated for a further 30 min, followed by three rounds of collagenase digestion at 37 °C for 20 min. The supernatant was collected and washed. Percoll gradient centrifugation was used to enrich LPMCs at the interface. Next, RNAiso Plus (Takara Bio Inc., Shiga, Japan) was used to extract mRNA from ear skin and colon LPMCs. RT-qPCR was performed using a SYBR Premix Ex Taq II kit (Takara Bio Inc., Shiga, Japan). The primer sequences used for RT-qPCR are listed in Table S1.

### PCR quantification of microbial fecal content

Intestinal fecal contents were obtained from WT and *clusterin*^−*/*−^ mice before and after IMQ administration (3 days). To examine the composition of the fecal microbiota, stool samples were sent to BioBankHealing (Sungnam, Korea). DNA was extracted and purified using a Maxwell^®^ RSC 48 (Promega, WI, USA). Stool samples were lysed, transferred to a prefilled reagent cartridge, and DNA was extracted and eluted. The microbial 16S rRNA gene was amplified (targeting the hyper-variable V3–V4 regions). The cycling conditions were as follows: initial denaturation of 94 °C for 3 min; followed by 25 cycles of denaturation at 94 °C for 45 s, annealing at 50 °C for 60 s, and extension at 72 °C for 5 min; and a final extension at 72 °C for 10 min. Sequencing was performed using an Illumina MiSeq platform (Illumina, CA, USA). The abundance of bacterial species was quantified using a Qubit (Thermo Fisher Scientific, MA, USA). Raw sequence data were analyzed using the QIIME2 microbiome analysis platform (https://qiime2.org).

### Human study population and skin specimen analysis

Patients with psoriasis and healthy controls who visited the Department of Dermatology, SMG-SNU Boramae Medical Center, Seoul, between 2011 and 2020 were enrolled in the study. All subjects gave informed consent for inclusion before participating in the study. The study was conducted in accordance with the Declaration of Helsinki, and the protocol was approved by the Ethics Committee and Institutional Review Board of SMG-SNU Boramae Medical Center (IRB No. 20-2019-34, 20-2019-45). The enrolled patients were previously diagnosed with psoriasis by qualified dermatologists. Skin samples were used to evaluate the severity of psoriasis, and patients were classified as mild or severe according to the grade of scale, erythema, and thickness. The human skin samples were fixed in formaldehyde and embedded in paraffin. Cut sections were immunostained with an anti-clusterin antibody (1:25; Abcam). Clusterin-positive areas (%) in the epidermis or dermis were analyzed using ImageJ software, which was calculated by averaging two random measurements (× 200).

### Statistical analysis

Data were analyzed using GraphPad Prism 7 (GraphPad Software, CA, USA) and SPSS software (version 25.0; SPSS Inc., IL, USA). The significance of differences between groups was determined using either the unpaired *t*-test or the Mann–Whitney U test. Spearman’s correlation analysis was used to determine the correlation between clusterin expression and psoriasis severity. Columns in the figures represent the mean ± SEM.

For the intestinal microbiota analysis, the Shannon’s index and Faith’s phylogenetic diversity index were used to assess the α-diversity of each group. Bacterial taxa that showed differential abundance by pairwise analysis of each group were identified using the Kruskal–Wallis nonparametric test. Weighted and unweighted UniFrac distance matrices were calculated for abundance and presence/absence data, respectively. A LDA model with an LEfSe was used to detect bacterial taxa showing significantly different abundances between groups (WT before IMQ vs*.* WT after IMQ; *clusterin*^−/−^ after IMQ vs. WT after IMQ). *P*-values < 0.05 were considered statistically significant (*P* < 0.001, *P* < 0.01, and *P* < 0.05 were marked with three, two, and one asterisks, respectively).

### Supplementary Information

Supplementary Figure S1.

Supplementary Figure S2.

Supplementary Figure S3.

Supplementary Figure S4.

Supplementary Information.

## Data Availability

The authors confirm that the data supporting the findings of this study are available within the article and its supplementary data. Raw data that support the findings of this study are available from the corresponding author, upon reasonable request.
