# Supplementary figures and images for: Regulation of psoriasis, colitis, and the intestinal microbiota by clusterin
Source: Sci Rep. 2023 Sep 16;13:15405. doi: 10.1038/s41598-023-42019-y (PMC10505212; doi:10.1038/s41598-023-42019-y)

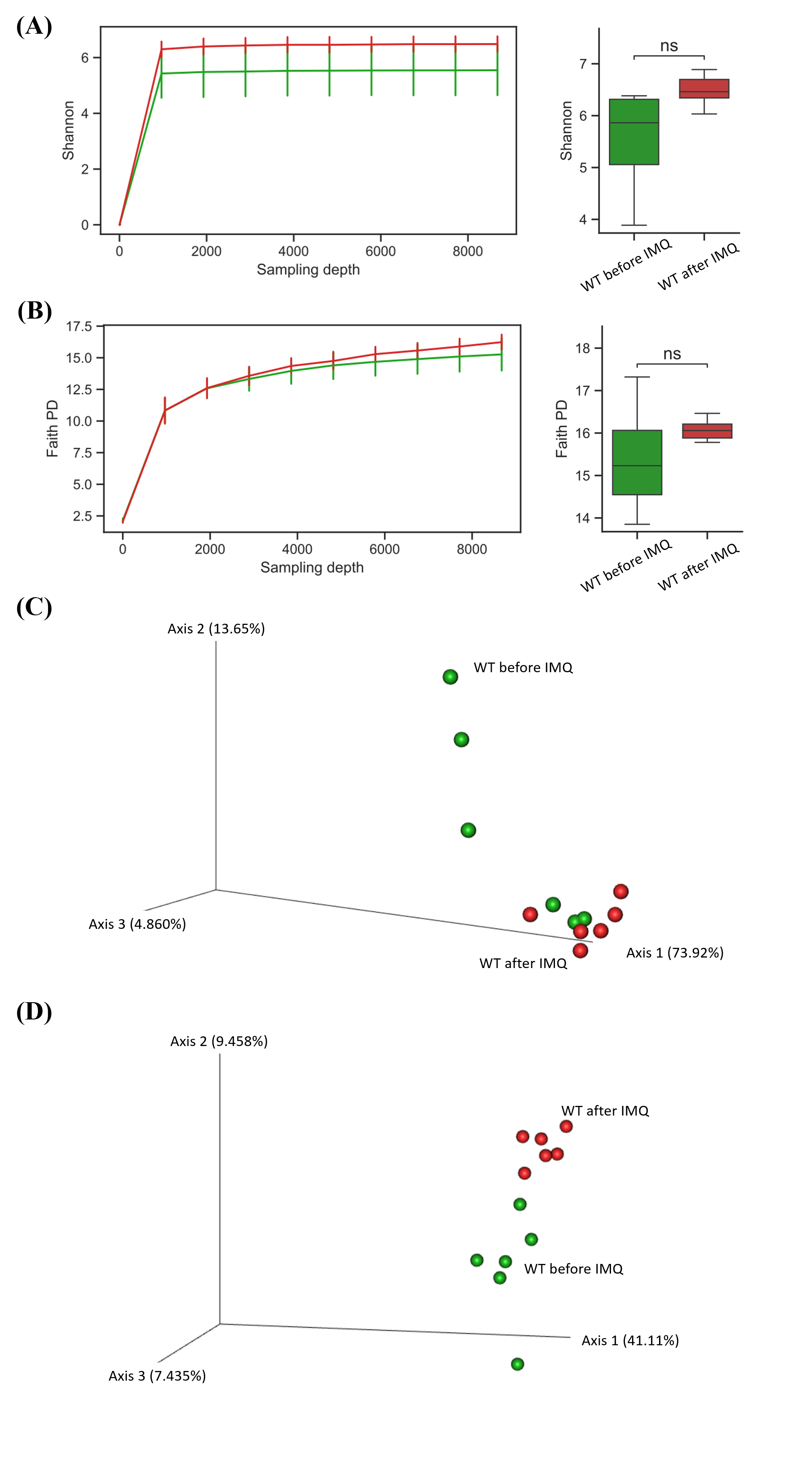

Supplement: Supplementary file 1 — Supplementary Figure S1. [file 41598_2023_42019_MOESM1_ESM.tif]

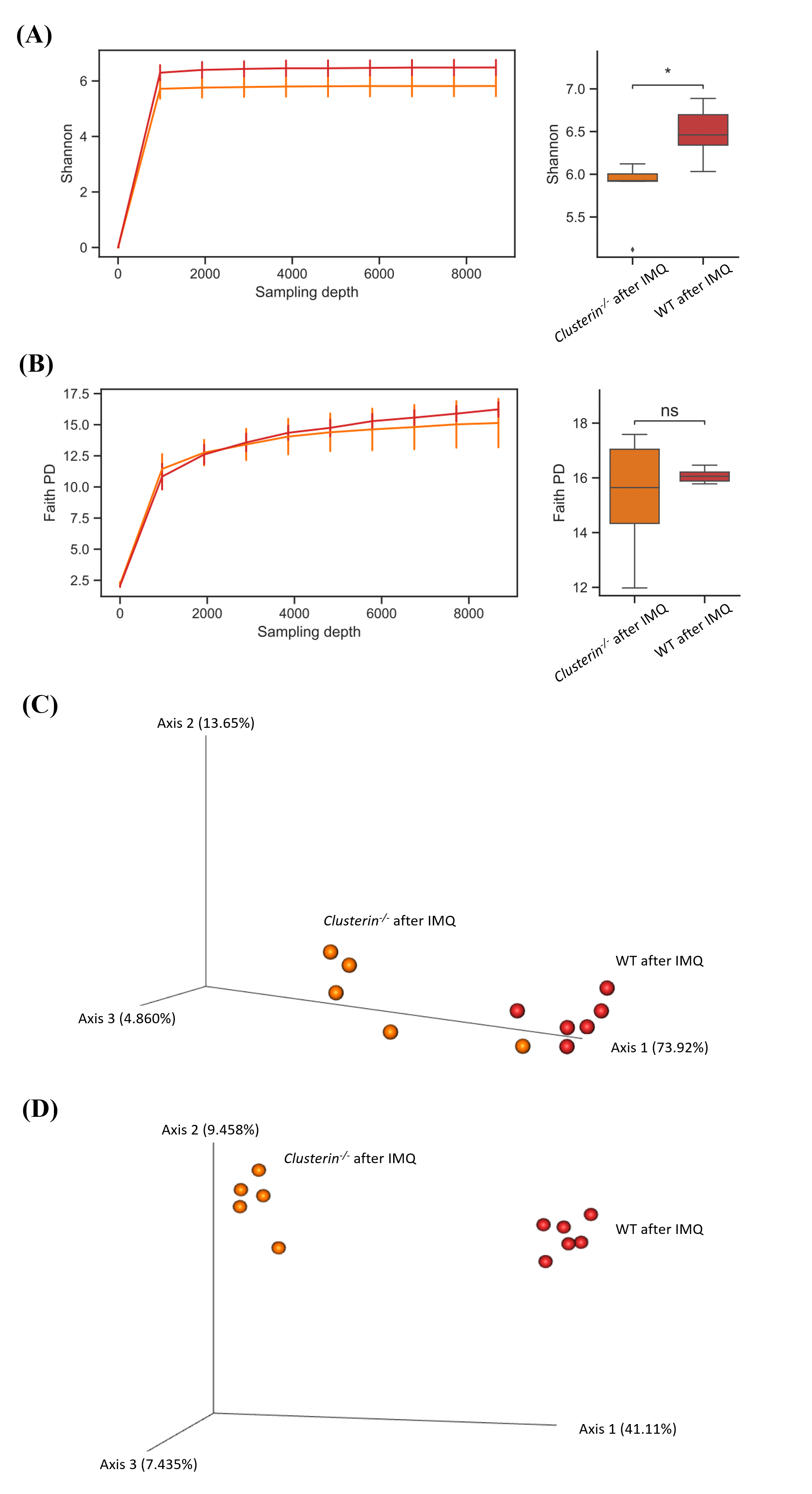

Supplement: Supplementary file 2 — Supplementary Figure S2. [file 41598_2023_42019_MOESM2_ESM.tif]

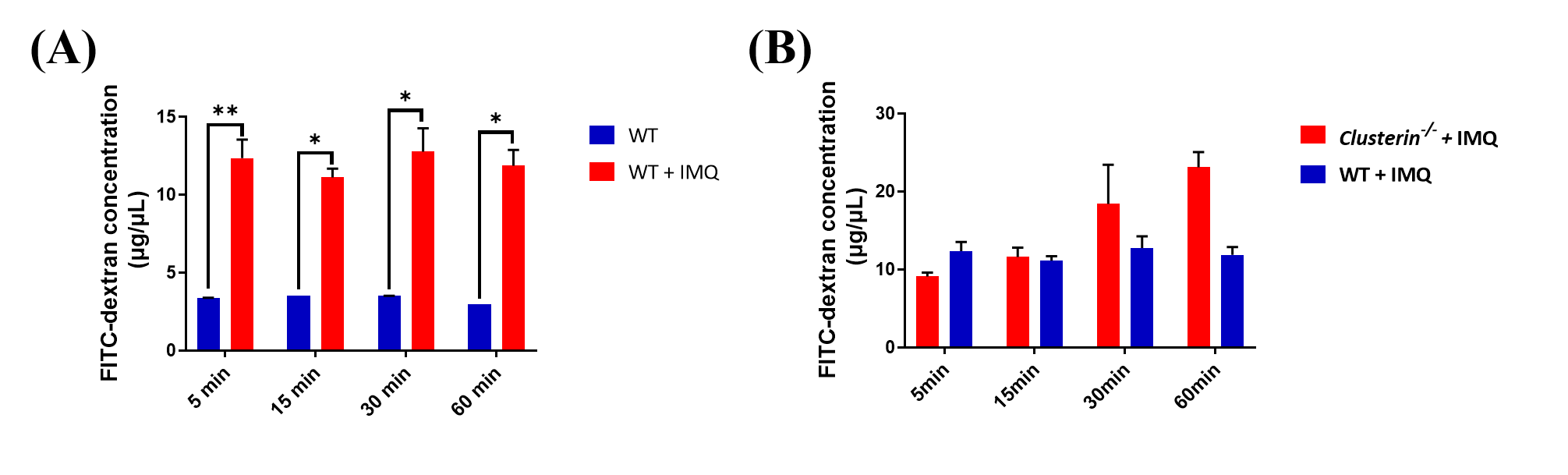

Supplement: Supplementary file 3 — Supplementary Figure S3. [file 41598_2023_42019_MOESM3_ESM.tif]

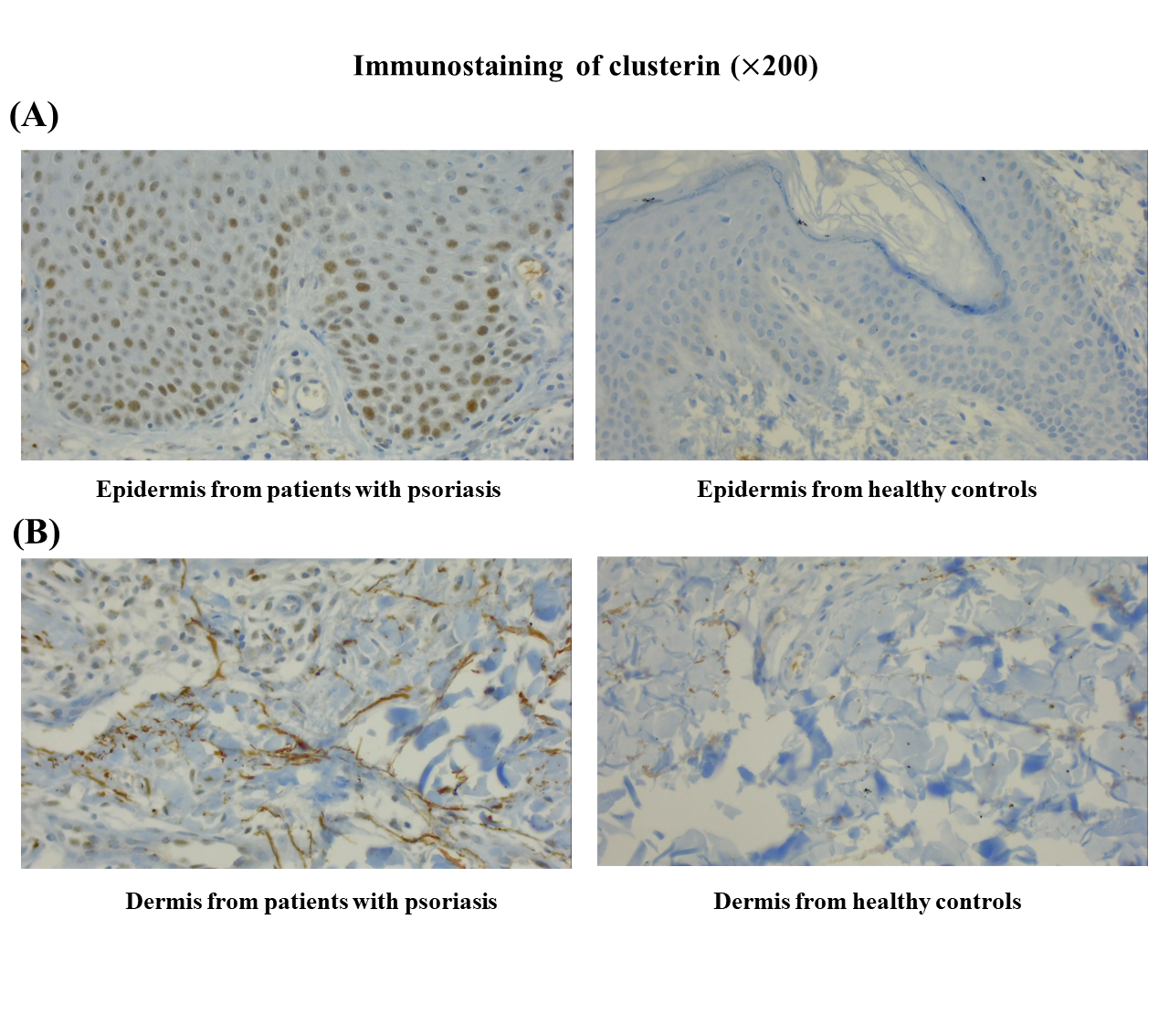

Supplement: Supplementary file 4 — Supplementary Figure S4. [file 41598_2023_42019_MOESM4_ESM.tif]
