# Supplementary material for: Regulation of psoriasis, colitis, and the intestinal microbiota by clusterin
Source: Sci Rep. 2023 Sep 16;13:15405. doi: 10.1038/s41598-023-42019-y (PMC10505212; doi:10.1038/s41598-023-42019-y)
Supplement: Supplementary file 5 — Supplementary Information. [file 41598_2023_42019_MOESM5_ESM.docx]

**Supplementary information**

**Supplementary Material**

**Assessment of intestinal permeability *in vivo***

After fasting for at least 4 h, WT and *clusterin^-/-^* mice were administrated 150 μg (180 mg/mL) FITC-dextran by oral gavage. Blood was collected at 5, 30, 45, 60, and 90 min and diluted 1:4 in phosphate-buffered saline. FITC-dextran was diluted with PBS at ratios of 1:4, 1:40, 1:125, 1:400, 1:1250, 1:4000, 1:12500, and 1:40000 to create a standard curve. The blood and standard curve samples were added to 96-well plates, and relative fluorescence units were measured using a spectrophotometer.

**Table S1.** Sequences of primers used for RT-qPCR.

| Mouse TNF-α | (F) 5′-CAT CTT CTA AAA ATC GAG TGA CAA-3′  (R) 5′-CAT CTT CTA AAA ATC GAG TGA CAA-3′ |
| --- | --- |
| Mouse IL-17 | (F) 5′-TCT CAT CCA GCA AGA GAT CC-3′  (R) 5′-AGT TTG GGA CCC CTT TAC AC-3′ |
| Human TNF-α | (F) 5′-CCC GAG TGA CAA GCC TGT AG-3′  (R) 5′-GAT GGC AGA GAG GAG GTT GAC-3′ |
| Human IL-1β | (F) 5′-CTT CAG CCA ATC TTC ATT GC-3′  (R) 5′-GTG GTC GGA GAT TCG TAG C-3′ |
| Human CXCL1 | (F) 5′-GCG CCC AAA CCG AAG TCA TA-3′  (R) 5′-ATG GGG GAT GCA GGA TTG AG-3′ |
| Human CCL20 | (F) 5′-TTG CTC CTG GCT GCT TTG-3′  (R) 5′-ACC CTC CAT GAT GTG CAA G-3′ |

**Supplementary figure legends**

**Figure S1.** Comparison of microbial composition between WT mice before and after IMQ treatment. (A) Shannon’s index. (B) Faith phylogenetic diversity index. (C) Weighted UniFrac distance. (D) Unweighted UniFrac distance. **P* < 0.05, ***P* < 0.01, ****P* < 0.001.

**Figure S2.** Comparison of microbial composition between IMQ-treated WT mice and *clusterin^-/-^* mice. (A) Shannon’s index. (B) Faith phylogenetic diversity index. (C) Weighted UniFrac distance. (D) Unweighted UniFrac distance. **P* < 0.05, ***P* < 0.01, ****P* < 0.001.

**Figure S3.** Relative permeability of the intestinal barrier to FITC-dextran. (A) Intestinal permeability in IMQ-treated WT mice was significantly higher than that in WT mice not treated with IMQ. (B) There was no significant difference in intestinal barrier function between IMQ-treated WT mice and *clusterin^-/-^* mice. **P* < 0.05, ***P* < 0.01, ****P* < 0.001.

**Figure S4.** Representative images of skin tissues from patients with psoriasis and healthy controls stained by immunohistochemistry using clusterin antibodies. (A) Epidermis from patients with psoriasis and healthy controls. (B) Dermis from patients with psoriasis and healthy controls.
